# Description of organizational and clinician characteristics of primary dementia care in Canada: a multi-method study

**DOI:** 10.1186/s12875-022-01732-9

**Published:** 2022-05-20

**Authors:** Mary Henein, Geneviève Arsenault-Lapierre, Nadia Sourial, Claire Godard-Sebillotte, Howard Bergman, Isabelle Vedel, Research on Organization of Healthcare Services for Alzheimer’s (ROSA) Team

**Affiliations:** 1 Lady Davis Institute for Medical Research, Jewish General Hospital, Montréal, QC 3755 Chemin de La Côte-Sainte-Catherine Canada; 2 Département de Médecine de famille et de Médecine d’urgence, Université de Montréal, Centre de Recherche du Centre Hospitalier de l’Université de Montréal (CHUM), 2900 Edouard Montpetit Blvd, Montreal, QC Canada; 3 Division of Geriatric Medicine, McGill University Health Centre, McGill University, 1001 Boulevard Décarie, Montréal, QC Canada; 4 International Affairs, Faculty of Medicine and Health Sciences, McGill University, 5858 chemin Côte-des-Neiges, Montréal, QC Canada; 5 Department of Family Medicine, McGill University, 5858 chemin Côte-des-Neiges, Montreal, QC Canada

**Keywords:** Health services organization, Dementia, Primary care, Quality of health care, Practice guidelines as topics

## Abstract

**Background:**

Organizational and clinician characteristics are important considerations for the implementation of evidence-based recommendations into primary care practice. The introduction of Canadian dementia practice guidelines and Alzheimer strategies offers a unique context to study which of the organizational and clinician characteristics align with good quality care in primary care practices.

**Methods:**

To evaluate the quality of dementia care, we carried out a retrospective chart review in randomly selected patients with a diagnosis of dementia and who had a visit during a 9-month period in 33 primary care practices. We collected data on indicators that were based on existing Canadian evidence-based recommendations to measure a quality of dementia care score. In addition, four questionnaires were administered: two questionnaires to evaluate the organizational characteristics of the practices (dementia-specific and general organization) and two to evaluate the clinician characteristics (one for family physicians and one for nurses). Primary care practices were stratified into tertiles based on their average quality of dementia care score (low, moderate, high). The differences between the groups organizational and clinician questionnaires scores were analyzed descriptively and visually.

**Results:**

The mean overall scores for each questionnaire were higher in the high quality of dementia care group. When looking at the breakdown of the overall score into each characteristic, the high-quality group had a higher average score for the dementia-specific organizational characteristics of “access to and coordination with home and community services”, “financial support”, “training”, “coordination and continuity within the practice”, and “caregiver support and involvement”. The characteristic “Leadership” showed a higher average score for the moderate and high-quality groups than the low-quality group. In both clinician questionnaires, the high group scored better in “attitudes towards the Alzheimer’s plan” than the other two groups.

**Conclusions:**

These results suggest that investing in organizational characteristics specifically aimed at dementia care is a promising avenue to improve quality of dementia care in primary care. These results may be useful to enhance the implementation of evidence-based practices and improve the quality of dementia care.

**Supplementary Information:**

The online version contains supplementary material available at 10.1186/s12875-022-01732-9.

## Contributions to the literature

- Organizational and clinician characteristics have been shown as important factors for implementation of evidence-based practice and may impact primary care practices’ adherence to dementia care recommendations.
- We found that dementia-specific organizational characteristics, including financing, leadership, and coordination within primary care clinic showed the widest differences in score between primary care practices grouped by quality of care..
- These characteristics should be considered for further study to determine their role in maximizing the uptake of evidence-based practice for dementia primary care

## Introduction

Evidence-based practice (EBP) is a key approach to improving the quality of patient care and service delivery in health care systems around the world, but it still faces implementation challenges in daily practice, in particular in primary care [1], which is also called the research-practice gap [2]. The implementation of evidence-based recommendations into daily practice is impacted by various factors, including organizational and clinician characteristics, that interplay and influence implementation effectiveness and sustainability [3, 4]. Organizational characteristics, such as team-based care, size of practice, and professional development have been shown to be important for effective disease management [5], especially for chronic diseases and depression [6, 7], improved healthcare delivery [8], and beneficial for health outcomes [9, 10], in particular for chronic illness [11]. Clinician characteristics are also important to consider when implementing evidence-based recommendations. Previous studies report that clinicians’ knowledge, attitudes, and practices serve as facilitators and barriers to implement evidence into practice [12–15].

Understanding the organizational and clinician characteristics that foster or hinder the introduction of evidence-based recommendations into daily practice is key [16, 17]. More studies on articulating the relationships among the organizational and clinician characteristics are needed [1].

In Canada, the dissemination of dementia clinical guidelines and implementation of dementia strategies offers unique and pertinent opportunities to better understand how organizational and clinician characteristics play a role in the quality of dementia care. Indeed, implementation of research findings into practice and guidelines has been at the forefront of improving the treatment and management of dementia in Canada [18, 19]. Since 1989, five Canadian dementia clinical guidelines have been disseminated [18, 20]. In addition, more recently, Provincial and National dementia strategies have been designed and implemented in Canada [21–25]. All these strategies have the same aim: to implement evidence-based practices in order to improve patient living with dementia (PLWD) access to care – in particular in primary care – enhance care continuity and empower patients and their caregivers.

These Canadian dementia guidelines and strategies prioritize the primary care setting, leveraging on team-based approach of primary care reforms (e.g., Family Health Teams/Organizations, Family Medicine Groups) [26, 27], with the support from secondary and tertiary care.

As dementia guidelines and strategies in primary care continue to be developed and implemented provincially and nationally, understanding which organizational and clinician characteristics within primary care facilitate or hinder better quality of dementia care is important. There are still gaps in knowledge when considering which organizational and clinician characteristics play a role in the quality of care provided by primary care practices for dementia care. Thus, the aim of this study was to describe organizational and clinician characteristics in primary care practices in Canada according to the primary care practice’s level of quality of dementia care.

## Methods

### Design and setting

We conducted an observational cross-sectional multi-method study as a part of a larger mixed methods study [28] in 33 purposively selected primary care practices in three Canadian provinces, namely, Ontario (ON), Québec (QC), and New Brunswick (NB). We used the STROBE reporting guidelines for cross-sectional studies (Additional file 1).

### Data sources

Five data sources were used: a retrospective chart review to measure the quality of dementia care score, two organizational questionnaires to measure dementia-specific organizational characteristics and general organizational characteristics, and two clinicians’ questionnaires to measure family physicians and primary care nurses’ Knowledge, Attitude, and Practice (KAP) characteristics related to dementia and dementia strategy.

### Quality of dementia care

To measure the quality of dementia care score, we performed a retrospective chart review [29]. We randomly selected the charts of 734 registered patients who were 75 years and older with a diagnosis of dementia and had a visit during one of two 9-month periods (October 1^st^ 2014-July 1^st^ 2015 or October 1^st^ 2015 – July 1^st^ 2016). The data collected was anonymized and included the presence or absence of documentation of 10 indicators, which were derived from current Canadian dementia guidelines [20, 30–32] and validated tools [33]. A score was determined based on the documented assessments of these indicators in the patient’s chart. Each participant was given a binary score depending on whether the indicator was assessed or not, according to their chart. These indicators were: assessment of cognitive testing, functional status, behavioural and psychological symptoms of dementia, weight, caregiver needs, driving status, home care needs, community service needs (e.g., Alzheimer Society), absence of anticholinergic medication and management of dementia medications. An overall quality of dementia care score was attributed to each patient as a proportion of the assessed indicators out of the eligible indicators. The average of the patients’ quality of dementia score was then attributed to each primary care practice.

### Organizational characteristics

To measure the dementia-specific and general primary care characteristics, we used two organizational questionnaires, described below. Both questionnaires were sent to each primary care practice’s medical director in one package with two copies of the consent form and a pre-stamped return envelope. Reminders were sent to increase response rate [28].

#### Dementia-specific organizational characteristics

To examine dementia-specific organizational characteristics, we used the validated Organizational Best Practices for Dementia Questionnaire [34]. It assesses nine dementia care specific organizational characteristics: “leadership”, “financial support”, “support from cognitive specialists”, “clinical information system”, “training”, “coordination and continuity within the clinic”, “caregiver support and involvement”, “access to and coordination with home and community services”, and “coordination with hospital”, and an overall score of Organizational Best Practices for Dementia. Each characteristic consisted of one or more questions related to that characteristic. The score for each characteristic was calculated by taking the mean of the responses for each question included in each characteristic and transforming it into a proportion of 100, where a higher score signified better alignment with good practices in dementia primary care. Missing responses were excluded from the characteristic score. The overall Organizational Best Practices for Dementia score is an average of the nine characteristics [34].

#### General primary care characteristics

To measure general primary care organizational characteristics, we adapted a validated questionnaire measuring an index of conformity to an ideal type (ICIT) of primary care [9, 35]. This questionnaire was developed for QC, then adapted and validated by our team for the two other provinces [28]. It assesses four characteristics: “vision” (e.g., shared values of the clinicians, organizational priorities), “resources” (e.g., number of clinicians and presence of nurses), “structure” (e.g., type of coordination of care, administrative responsibilities, type of financing) and “practice” (e.g., availability for urgent care, availability of specialty services, primary consultation type), which contribute to the overall ICIT score. For our study, we report the score for each characteristic, which was calculated by taking the sum of the responses in each item of the given characteristics and then calculating the proportion out of the maximum possible points. The overall ICIT score was calculated by summing the characteristics and taking the proportion of the maximum eligible points. If a question was left blank, this was excluded from the eligible points. For each characteristic and the overall ICIT score, a higher score means a higher conformity to An Ideal Type of Primary Care [35].

#### Clinician characteristics

To measure the KAP of primary care clinicians towards dementia, two questionnaires (one for physicians and the other for nurses) were designed and validated by our team [36, 37]. Questionnaires were sent in a personalized package to each physician and nurse within each practice, which included a consent form, the questionnaire, and a letter explaining the questionnaire [28].

#### Physicians’ knowledge, attitudes, and practices toward dementia

The physician questionnaire assessed five characteristics: “perceived knowledge and competence”, “practice with regard to evaluation”, “attitudes towards dementia”, “collaboration with nurses”, and “attitudes towards the Alzheimer plan”, as well as an overall physician score. Each characteristic score was calculated by taking the mean of the responses to the Likert-scale items (scored from 1–4 or 1–10) within that characteristic and translating it to a scale of 100, excluding missing responses. The overall physician score was calculated by taking the mean of the characteristic scores. A higher score indicated better KAP towards dementia [37].

#### Nurses’ knowledge, attitudes, and practice toward dementia

The nurse questionnaire assessed four characteristics: “perceived knowledge and competence”, “attitudes towards patients and caregivers”, “perceived support from the community”, and “attitudes towards the Alzheimer’s plan”, as well as an overall nurse score was calculated. Each characteristic score was calculated by taking the mean of the responses to the Likert-scale items (scored from 1–4 or 1–10) within that characteristic and transforming it to a scale of 100, excluding missing responses. An overall nurse score was calculated by taking the mean of the characteristic scores. A higher score indicated better KAP towards dementia [36].

### Statistical analysis

Due to the number of comparisons and limited sample size, statistical analysis was descriptive. Data was aggregated by primary care practice. Practices were then divided into tertiles based on their mean quality of dementia care score: low- (below 33^rd^ percentile), moderate- (between 33^rd^ and 67^th^ percentile) and high- (higher than 67^th^ percentile) quality of dementia care score. The means and standard deviations (SD) of the overall and specific characteristics of organizational and clinician characteristics was calculated for each quality of dementia care group (low-, moderate-, high-quality). We determined how many practices did not return each questionnaire.

We used radar plots to visually determine differences in the organizational and clinician characteristic according to quality of dementia care group. Radar plots allow for a multivariate data in a two-dimensional graph [38]. Four radar plots were created for the four groups of characteristics (dementia organizational characteristics, general primary care organizational characteristics, physician characteristics and nurse characteristics) using their mean characteristics scores. Each radar plot has three lines representing the scores from the low-, moderate-, and high-quality groups which are overlaid onto one plot where each node represents a specific characteristic. The axis starts in the center (zero) and increases as it reaches the circumference of the plot (100).

### Ethics

This study was approved by the Research Ethics Board (REB) at Centre Intégré Universitaire de Santé et de Service Social (CIUSSS) du Centre-Ouest-de-l'île-de-Montréal and from each Centre Intégré de Santé et de Service Social or CIUSSS involved in Québec, from the REB at the University of Waterloo, and from the REB from Université de Moncton and both regional health boards in New Brunswick [28]. Access to patients’ medical chart data was granted upon institutional board of each CISSS or CIUSSS. Questionnaire were sent to each practice or clinician along with a consent form, and only those with a returned with a written, signed consent form were included in analysis.

## Results

This study was conducted among 33 Canadian primary care practices: 17 practices in QC, eight in ON, and eight in NB.

### Quality of dementia care score

The mean overall quality of dementia care score across the primary care practices was 45.3 (Standard deviation (SD) = 15.9, range = [0.0, 64.6]). Practices were divided into the low-, moderate-, or high-quality group based on their quality of dementia care score. Each quality group had 11 primary care practices. The quality of dementia care scores in the low-quality group ranged from 0 to 37.3, that of the moderate-quality group ranged from 40.4 to 57.3, and that of the high-quality group ranged from 57.4 to 64.6 (Table 1).

**Table 1:** Demographic information

	Low QOC (*n* = 11)	Moderate QOC (*n* = 11)	High QOC (*n* = 11)	Overall (*n* = 33)
Quality of dementia score, mean (SD)	26.9 (10.7)	48.2 (6.0)	60.8 (2.2)	45.3 (15.8)
Quality of dementia score, range	0.0–37.3	40.4–57.3	57.4–64.6	0.0–64.6

*QOC* Quality of care score, *N* Number of practices, *SD* Standard deviation

### Organizational characteristics

A total of 30 primary care practices returned a completed dementia-specific organizational questionnaire and a completed general primary care characteristics questionnaire.

#### Dementia-specific organizational characteristics

**Fig. 1:**
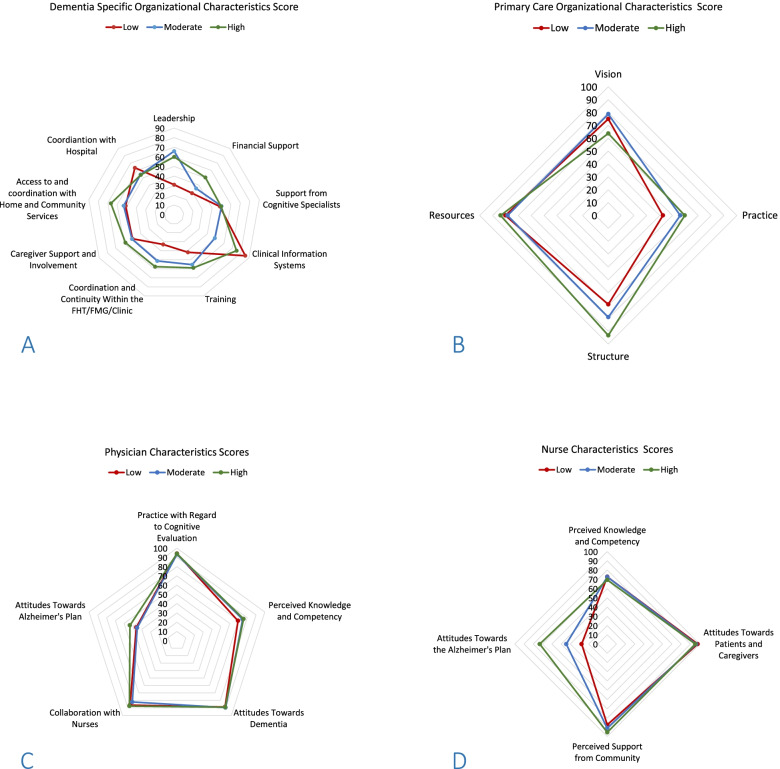
Radar plots for each questionnaire evaluating the organizational and clinical characteristics. **1A** Organizational scores from Dementia Specific Organizational Characteristics Questionnaire. Footnote: The axis increases from the center of the axis (zero) to the circumference of the plot (100). Higher scores signify better alignment with good practices in dementia primary care. **1B** Organizational scores from Primary Care Organizational Characteristics Questionnaire. Footnote: The axis increases from the center of the axis (zero) to the circumference of the plot (100). A higher score means a higher conformity to An Ideal Type of Primary Care. **1C** Clinician scores from the physician questionnaire. Footnote: The axis increases from the center of the axis (zero) to the circumference of the plot (100). A higher score indicated higher physician KAP towards dementia. **1D** Clinician scores from the nurse questionnaire. Footnote: The axis increases from the center of the axis (zero) to the circumference of the plot (100). A higher score indicated higher nurse KAP towards dementia

The mean overall dementia-specific organizational characteristics score was 59.0 (SD = 12.6) in the high-quality group, compared to 47.7 (SD = 12.2) in the low-quality group (Table 2 and Additional file 2). In particular, scores related to access to and coordination with home care, caregiver support, coordination within primary care practice, financial support, and training were higher in the high-quality group compared to the moderate and low-quality group (Additional file 2 and Fig. 1A). Leadership was also considerably higher in the high and moderate care groups than the low-quality group.

**Table 2:** Mean scores for each questionnaire grouped by quality of dementia care score

	Low Score group	Moderate Score group	High Score group	Overall
Dementia-specific organizational score, mean (SD), *n* = 30; 3 missing	47.7 (12.2)	51.9 (12.4)	59.0 (12.6)	52.9 (12.9)
General primary care organization score (ICIT), mean (SD), *n* = 30; 3 missing	63.2 (14.8)	70.0 (16.0)	72.2 (8.5)	68.5 (13.6)
Overall score physicians, mean (SD), *N* = 30; 3 missing	76.9 (6.4)	77.0 (6.8)	79.9 (3.7)	78.1 (5.7)
Overall score nurses, mean (SD), *n* = 31; 2 missing	70.5 (10.7)	74.4 (13.0)	83.4 (4.9)	76.3 (11.2)

*QOC* Quality of care score, *ICIT* Index of Conformity to an Ideal Type, missing represents the number of missing full questionnaires

#### General primary care characteristics

The mean overall general primary care organizational scores were 63.2 (SD = 14.8) in the low-quality group; 70.0 (SD = 16.0) in the moderate-quality group; 72.2 (SD = 8.5) in the high-quality group (Table 2 and Additional file 2). The high-quality group had a higher average score in the characteristics’ “structure” and “practice”, while “resources” was similar among the three groups (Additional file 2 and Fig. 1B).

### Clinician characteristics

Family physician questionnaires and nurse questionnaires were returned from 30 and 31 clinics, respectively.

#### Physician knowledge, attitude, practice toward dementia

The practice’s mean overall scores for the physician questionnaire was similar between high, moderate, and low score groups: 79.9 (SD = 3.7), 77.0 (SD = 6.8), and 76.9 (SD = 6.4), respectively (Table 2). Specifically, the results of these three quality groups were similar for the characteristics “practice” with regard to cognitive evaluation and attitudes towards dementia, “attitudes towards dementia”, “perceived knowledge and competency” and “collaboration with nurses” (Additional file 2 and Fig. 1C). Scores were slightly higher for the high-quality group in the characteristics “attitudes towards the Alzheimer’s plan”, compared to the moderate and low-quality groups (Additional file 2 and Fig. 1C).

#### Nurse knowledge, attitude, practice toward dementia

The practice’s mean overall score for the nurse questionnaire of the high-quality group was 83.4 (SD = 4.9), 74.4 (SD = 13.0) for the moderate-quality group, and 70.5 (SD = 10.7) for the low-quality group (Table 2). In particular, the largest difference between the quality groups was observed for the characteristic “attitudes towards the Alzheimer Plan”.

## Discussion

Our study uniquely examines organizational and clinician characteristics of primary care practices in Canada and how these characteristics interplay with different levels of quality of dementia care. We observed overall better organizational and clinician scores in primary care practices with high-quality dementia care. Primary care practices that were categorized in higher-quality dementia care groups had higher overall general primary care organization, dementia-specific organization, and physician and nurse KAP scores.

In terms of organizational characteristics, we identified the largest differences between quality of care groups for “financial support”, “leadership”, and “coordination within FHT/FMG/Clinic”. In terms of clinician characteristics, we found that “attitudes towards the Alzheimer’s plan” showed the largest difference between the quality of care groups among both clinicians, especially for nurses.

Our results align with recent literature that reported physicians perceived financial support as an important aspect for improving the quality of primary dementia care [39]. A systematic review also found that leadership and service funding was important for the feasibility of primary care dementia initiatives [40]. Furthermore, dementia-specific programs or interventions that aimed at coordination and continuity within the practice have demonstrated some improved outcomes for PLWD [29, 41]. Institutional support (including financial support, interdisciplinary care, and training) was demonstrated to be associated with higher quality of care in a previous study from our team [42].

The results from our present study suggest that dementia-specific organizational characteristics should be the focus of future research on the quality of dementia care, such as the impact of dedicated financial support for dementia provided to primary care practices, improvement of coordination and continuity of care within the primary care practice and having leadership within the primary care clinic for dementia.

We found that physicians’ and nurses’ attitudes towards dementia, and physicians’ practice (with regard to cognitive evaluation) were similar between each quality of dementia care groups. The overall high average scores and lack of variation for these characteristics could be explained by a ceiling effect, suggesting that physicians feel confident in their abilities to conduct cognitive evaluations. While these results are at risk of biases such as acquiescence response bias or selection bias, other recent studies have found family physicians are confident in their initial management of dementia [43], agree on the role of primary care in identifying dementia and believe there is much that can be done to improve the lives of those living with dementia [44]. In particular, physicians that were provided specific training had improved clinician perceptions and attitudes towards dementia care [45]. This is a shift from previous studies, which found that family physicians felt unequipped or not confident in caring for those with dementia [46, 47]. Thus, family physicians may receive more or better training than previously, and this may explain the high scores for clinician characteristics, such as attitudes towards dementia and practice, across all quality groups.

### Strengths and limitations

This study was a coordinated undertaking between three Canadian provinces and thus gathers data from a wide variety of settings. Also, these provinces were at different stages of implementing dementia strategies, allowing a diverse outlook on primary care practices with different support for dementia care.

Our study, however, has some limitations. This study is at risk of response bias because the data collected is based on self-reported questionnaires. However, our validated quality of dementia care score was independently measured in patients’ chart by researchers outside the primary care practice. The quality groups (low, moderate, high) were not predetermined; rather, we used tertiles to create the quality groups to make them comparable relative to one another. Finally, while our results are descriptive in nature and cannot infer association, they allow us to observe a wide range of key organizational and clinician characteristics and generate hypotheses on how these characteristics interplay to explain the observed differences in the quality of dementia care across a variety of primary care practices in Canada.

## Conclusion

In describing the organizational and clinician characteristics and quality of dementia care for primary care practices in Canada, our study provides a renewed perspective on avenues to improve dementia primary care. In particular, policy-makers, managers and clinicians should consider organizational characteristics that are designed specifically with dementia management in mind to support uptake of evidence in daily practice and improve the quality of dementia care in primary care.

## Supplementary Information

**Additional file 1. **STROBE checklist

**Additional file 2.** Questionnaire scores at the practice level and grouped by quality-of-care score

## Data Availability

The datasets generated and/or analysed during the current study are not publicly available due confidentiality but anonymized aggregated data are available upon reasonable request to the corresponding author.

## Abbreviations

EBP: Evidence-Based Practice
ON: Ontario
QC: Québec
NB: New Brunswick
KAP: Knowledge, Attitudes and Practice
PLWD: Persons living with Dementia
ICIT: Index of Conformity to an Ideal Type
