# Supplementary material for: Description of organizational and clinician characteristics of primary dementia care in Canada: a multi-method study
Source: BMC Prim Care. 2022 May 20;23:121. doi: 10.1186/s12875-022-01732-9 (PMC9121549; doi:10.1186/s12875-022-01732-9)
Supplement: Supplementary file 2 — Additional file 2. Questionnaire scores at the practice level and grouped by quality-of-care score [file 12875_2022_1732_MOESM2_ESM.pdf]

Additional File 2: Questionnaire scores at the practice level and grouped by quality of care score

Table 1: Dementia-Specific Organizational Characteristics scores across primary care practices (N= 30)

Characteristic	Mean	SD
Leadership	52.3	24.2
Financial Support	38.4	27.5
Support from Cognitive Specialists	49.4	15.1
Clinical Information Systems	69.3	25.9
Training	52.0	16.9
Coordination and Continuity Within the FHT/FMG/Clinic	47.3	20.4
Caregiver Support and Involvement	53.0	23.9
Access to and coordination with Home and Community Services	57.1	12.3
Coordination with Hospital	57.3	22.2
Overall Score	52.9	12.9

Table 2: General Primary Care Characteristics scores across primary care practices (N= 30)

Characteristic	Mean	SD
Vision	72.5	18.8
Practice	52.7	19.5
Structure	80.6	24.3
Resources	80.7	16.7
Overall Score	68.5	13.6

*Scores are expressed on 100.

Table 3: Organizational and clinician Characteristics scores by quality of care (QOC) groups (N = 30)

	LOW QOC Score group		MODERATE QOC Score group		HIGH QOC Score group	
	Mean	SD	Mean	SD	Mean	SD
**Overall Score**	47.7	12.2	51.9	12.4	59.0	12.6
Leadership	31.0	23.8	66.0	14.3	60.0	18.3
Financial Support	29.0	22.9	35.6	25.8	50.5	31.0
Support from Cognitive Specialists	48.4	14.9	50.1	17.1	49.7	14.9
Clinical Information Systems	85.6	22.4	49.0	23.3	75.0	18.4
Training	41.8	16.6	55.4	16.8	59.0	13.3
Coordination and Continuity Within the Primary Care Practice	33.0	18.6	51.2	14.2	57.6	20.8
Caregiver Support and Involvement	50.0	30.5	50.6	19.7	58.3	21.8

Access to and Coordination with Home and Community Services	51.3	9.0	53.3	12.7	66.8	9.4
Coordination with Hospital	63.6	16.8	54.4	27.5	54.0	21.9
**Part B**						
**Overall Score**	63.2	14.8	70.0	16.0	72.2	8.5
Vision Score	75.0	17.7	78.8	15.0	63.8	21.6
Practice Score	42.5	21.8	56.0	18.6	59.5	15.2
Structure Score	69.2	31.4	79.2	22.7	93.3	8.6
Resources Score	80.0	14.7	78.3	25.1	83.8	6.0

Table 4: Family Physician Knowledge, Attitudes and Practices scores across primary care practices (N=30)

**Factor**	**Mean**	**SD**
Practice with Regard to Cognitive Evaluation	93.8	3.6
Perceived Knowledge and Competency	73.8	6.0
Attitudes Towards Dementia	89.1	4.0
Collaboration with Nurses	85.1	10.7
Attitudes Towards the Alzheimer Plan	48.5	17.3
**Overall Score Physicians**	78.1	5.7

Table 5: Nurse Knowledge, Attitudes and Practices scores across primary care practices (N=30)

**Factor**	**Mean**	**SD**
Perceived Knowledge and Competency	71.5	10.6
Attitudes Towards Patients and Caregivers	96.4	3.5
Perceived Support from The Community	92.0	10.2
Attitudes Towards the Alzheimer Plan	49.1	29.9
**Overall Score Nurses**	76.3	11.2

Table 6: Family Physician Knowledge, Attitudes and Practices scores by quality of care (QOC) groups (N=30)

	**LOW QOC Score group**		**MODERATE QOC Score group**		**HIGH QOC Score group**	
	**Mean**	**SD**	**Mean**	**SD**	**Mean**	**SD**
Practice with Regard to Cognitive Evaluation	94.2	5.0	93.3	3.7	94.0	2.6
Perceived Knowledge and Competency	69.5	6.3	74.8	5.1	75.9	5.6
Attitudes Towards Dementia	88.7	4.7	89.5	4.7	88.9	2.8

Collaboration with Nurses	86.2	10.3	82.1	14.3	87.4	6.4
Attitudes Towards the Alzheimer Plan	46.2	16.2	45.2	21.8	53.5	12.7
**Overall Score Physicians**	76.9	6.4	77.0	6.8	79.9	3.7

Table 7: Nurses Knowledge, Attitudes and Practices scores by quality of care (QOC) groups (N=31)

	**LOW QOC Score group**		**MODERATE QOC Score group**		**HIGH QOC Score group**	
	**Mean**	**SD**	**Mean**	**SD**	**Mean**	**SD**
Perceived Knowledge and Competency	72.7	12.1	72.6	10.8	69.4	9.7
Attitudes Towards Patients and Caregivers	97.9	2.9	96.2	3.6	95.3	3.8
Perceived Support from the Community	87.4	14.8	91.1	11.0	95.5	3.8
Attitudes Towards the Alzheimer Plan	27.6	21.5	44.4	33.4	73.0	11.4
**Overall Score Nurses**	70.5	10.7	74.4	13.0	83.4	4.9
